# Blood glucose, diabetes and metabolic control in patients with community-acquired pneumonia

**DOI:** 10.1007/s00125-020-05225-1

**Published:** 2020-07-17

**Authors:** Philipp M. Lepper, Robert Bals, Peter Jüni, Maximilian von Eynatten

**Affiliations:** 1 Department of Internal Medicine V – Pneumology, Allergology and Intensive Care Medicine, University Hospital of Saarland, Kirrberger Strasse 100, 66421 Homburg/Saar, Germany; 2 Applied Health Research Centre (AHRC), Li Ka Shing Knowledge Institute of St. Michael’s Hospital, Toronto, ON Canada; 3 Department of Medicine and Institute of Health Policy, Management and Evaluation, University of Toronto, Toronto, ON Canada; 4 Nestlé Health Science, Vevey, Switzerland

**Keywords:** Community-acquired pneumonia, COVID-19, Diabetes mellitus, Hyperglycaemia

*To the Editor:* We read with interest the studies by Cariou et al [1] and Wang et al [2] published in the journal. Evidence so far suggests that type 2 diabetes mellitus may not increase the overall risk of an infection with the novel coronavirus severe acute respiratory syndrome coronavirus 2 (SARS-CoV2) [3], the causal agent of a syndrome called coronavirus disease-2019 (COVID-19); however, pre-existing type 2 diabetes appears to be associated with more severe infection [4]. Moreover, recent evidence by Zhu et al [5] and data from an observational NHS England cohort [6, 7], consistently showed an increased risk for COVID-19-related death in patients with type 2 diabetes. The study by Cariou et al [1] adds to current knowledge by reporting that higher BMI (but not long-term glycaemic control or type of glucose-lowering therapy used) was significantly associated with the severity of COVID-19 infection as studied in their cohort of 1317 patients with pre-existing diabetes (89% with type 2 diabetes). Wang and colleagues analysed whether blood glucose taken within 24 h of admission after a fasting period was a predictive marker for 28-day mortality in 605 COVID-19 patients without diabetes [2]. They found that patients with fasting serum glucose concentrations ≥7.0 mmol/l had a significantly higher risk of death (HR 2.30; 95% CI 1.49, 3.55; *p* = 0.0002). Hence, currently available evidence on clinical risk management in patients with COVID-19 strongly supports the inclusion of assessment of diabetes status as well as careful consideration of additional clinical attributes, such as BMI and blood glucose levels.

These important insights may have just recently emerged for COVID-19; however, previous evidence in clinical community-acquired pneumonia (CAP) research has already identified the importance of pre-existing diabetes for outcome risk assessment. We conducted a large prospective study in 6891 Caucasian patients admitted for CAP due to causes other than SARS-CoV2 to 12 German centres [8]. We explored whether pre-existing diabetes would be associated with an increased mortality risk from CAP caused by any infectious agent [8]. The 28-day mortality risk was significantly increased in the 1114 participants with diabetes compared with that in individuals without diabetes with normal serum glucose levels (adjusted HR 1.92; 95% CI 1.34, 2.75; *p* < 0.001).

Notably, evidence for assessing COVID-19 mortality risk has recently expanded beyond exploring diabetes status and indicated that assessing blood glucose levels could further identify at-risk individuals for adverse outcomes. Cariou and colleagues report an age- and sex-independent association between increased admission plasma glucose levels and the severity of COVID-19, as well as early mortality at day 7, among individuals with diabetes [1]. In our previous study we also utilised admission glucose values to further categorise patients into normo- and hyperglycaemic subgroups [8]. This glucose-based stratification allowed us to identify individuals at further increased risk for CAP mortality. Compared with participants with normal plasma glucose levels and without diabetes at admission, the adjusted HR for death within 28 days after admission in those with diabetes was 1.92 (95% CI 1.34, 2.75; *p* < 0.001) and the observed mortality risk remained largely unchanged for up to 180 days of follow-up [8].

Importantly, the clinical utility of glucose levels on admission appears to extend to CAP patients without pre-existing diabetes. The study by Wang et al [2], as well as our previous study [8], found a significant association between admission glucose and CAP mortality outcome in individuals without known diabetes. We reported that glucose levels on admission in patients admitted for CAP but without pre-existing diabetes (*n* = 5141) were significantly associated with all-cause mortality. In non-diabetic individuals 28- and 90-day mortality was increased whether glucose concentrations were assessed by a specific glucose threshold level (i.e. ≥6.0 mmol/l on admission; HR 1.71; 95% CI 1.22, 2.40; *p* for trend 0.001) or were categorised in a stepwise manner (i.e. <4 mmol/l, 4–5.99 mmol/l [reference group], 6–10.99 mmol/l [HR 2.89; 95% CI 2.27, 3.69], 11–13.99 mmol/l [HR 4.01; 95% CI 2.78, 5.81], and ≥14 mmol/l [HR 6.04; 95% CI 4.18, 8.74], all *p* for trend <0.001). Interestingly, in our study, the almost threefold mortality risk in individuals without diabetes but with elevated glucose levels on admission within the moderate range (6–10.99 mmol/l) was comparable to the HR for death at 28 days seen for individuals with COVID-19 and an FBG ≥7.0 mmol/l as reported by Wang et al of 2.30 (95% CI 1.49, 3.55) [2].

Thus, the available evidence shows that mortality risk from CAP (including COVID-19) is considerably increased in individuals with pre-existing diabetes, but also under conditions of acutely elevated blood glucose levels, even in patients without previously diagnosed diabetes. Based on these results, clinical risk assessment based on pre-existing diabetes status in individuals with COVID-19 is essential. If diabetes is present, BMI, as well as rigorous and serial assessments of in-hospital glucose levels (defined as at least one documented 2 h post-prandial glucose value exceeding 10 mmol/l) could add further value for risk stratification [5]. However, it may be difficult to obtain this information within a useful timeframe for the latter, and we suggest that a single point-of-care measurement of glucose, especially at standardised timepoints (i.e. at hospital admission without a fasting period) could be an attractive alternative. Notably, hospital admission glucose may be of particular importance for people presenting with COVID-19 without pre-existing diabetes, as this clinical marker could identify at-risk individuals that may easily be missed by solely assessing diabetes status.

In summary, evidence from large CAP cohorts (including patients with and without COVID-19) from different geographical regions and ethnic groups, strongly suggests that individuals with pre-existing diabetes and/or elevated blood glucose levels need special attention when presenting to a hospital, as they represent a group at high risk for increased mortality (Table 1). We believe admission glucose could represent an attractive clinical tool for early and effective CAP risk assessment as it is readily available in daily practice.

**Table 1:** HRs for selected groups of patients with CAP, including COVID-19

Study	Population	Preexisting diabetes	Patients (*n*)	Glucose level (mmol/l)	OR or HR (95% CI) for death	*p* value
Cariou et al [1]	COVID-19	Yes	1317	>5.55	Increased for death on day 7	0.0059
Zhu et al [5]	COVID-19	Yes	952	Any	HR 2.90 (2.21, 3.81)	<0.001
Lepper et al [8]	CAP	Yes	1114	Any	HR: 1.92 (1.34, 2.75)	<0.001
		No	5756	≥6.0	HR: 1.69 (1.24, 2.30)	<0.001
Wang et al [2]	COVID-19	No	605	≥7.0	HR: 2.30 (1.49, 3.55)	0.0002

## Abbreviations

CAP: Community-acquired pneumonia
COVID-19: Coronavirus disease-2019
SARS-CoV2: Coronavirus severe acute respiratory syndrome coronavirus 2
